# Eating Disorders and Metabolic Diseases

**DOI:** 10.3390/ijerph20032446

**Published:** 2023-01-30

**Authors:** Zhiping Yu, Valerie Muehleman

**Affiliations:** 1 Department of Nutrition and Dietetics, University of North Florida, 1 UNF Drive, Jacksonville, FL 32224, USA; 2 Beaufort Jasper Hampton Comprehensive Health Services, Inc., P.O. Box 357, Ridgeland, SC 29926, USA

**Keywords:** eating disorders, obesity, diabetes, metabolic syndrome

## Abstract

Eating disorders are complex diseases with multifactorial causes. According to the *Diagnostic and Statistical Manual of Mental Disorders text version (DSM-5-TR)* and the *WHO International Classification of Diseases and Related Health Problems (ICD-11)*, the major types of eating disorders include anorexia nervosa, bulimia nervosa, and binge eating disorder. The prevalence of eating disorders is alarmingly increasing globally. Moreover, the COVID-19 pandemic has led to more development and worsening of eating disorders. Patients with eating disorders exhibit high rates of psychiatric comorbidities and medical comorbidities such as obesity, diabetes, and metabolic syndrome. This paper aims to review and discuss the comorbidities of eating disorders with those metabolic diseases. Eating disorder treatment typically includes a combination of some or all approaches such as psychotherapy, nutrition education, and medications. Early detection and intervention are important for the treatment of eating disorders.

## 1. Introduction

Eating disorders are health problems that were first reported in the 17th century but dramatically increased in the late 20th century. They initiated in Western countries and evolved, rapidly catching the attention of both Western and non-Western countries nowadays. Prior to the 20th century in America, the “ideal” body shape of a young woman was a full-figured woman. By the late 1960s, women had lost much of their curves [1]. The media constantly present us with images of the “ideal” body shape, for both men and women. As a result, many young females are inspired to be thin; some young men feel they need to lift weights excessively to increase their muscle mass. These types of messages and social pressure may cause body dissatisfaction, extreme dieting, and unhealthy weight control methods, which eventually lead to the development of eating disorders [2].

According to a 2019 systematic review paper, over the time period between 2000 and 2018, the point prevalence of all eating disorders increased from 3.5% in 2000–2006 to 4.9% in 2007–2012, and 7.8% for the 2013–2018 period [3]. The anomality has more than doubled. During the 2013–2018 period, the point prevalence was 8.8% in adults and 5.7% in adolescents [3]. In addition, numerous studies did not distinguish between adults and adolescents; thus, those studies were analyzed as a “mixed” category, which is about 8.5% [3]. This indicates that eating disorders are highly prevalent in adolescents already, which is consistent with many other studies reporting the early onset of eating disorders [4]. Specifically, the median age of onset was reported to be 21 years old for binge eating disorder and 18 years old for anorexia and bulimia nervosa [5].

Eating disorders can affect people of all genders, ages, races, ethnicities, body shapes, weights, sexual orientations, and socioeconomic statuses. Females have a higher prevalence than males (2–3 times higher) [6], possibly due to greater body dissatisfaction and higher tendency to experience depression, stress, and anxiety than males [7]. More importantly, eating disorders can cause a wide variety of health problems such as obesity, type-2 diabetes, hypertension, high cholesterol, heart disease, gallbladder disease, or even death [8,9]. The cost of eating disorders has been estimated to equate to USD 11,808 per person [10]. Fortunately, with treatment, 60% of patients can fully recover [11]. However, only about half of people with an eating disorder will seek and receive treatment [12]. Early detection and intervention are important.

This paper will provide an updated overview of eating disorders and focus on discussing the comorbidities of eating disorders and metabolic diseases.

## 2. Types and Diagnoses of Eating Disorders

As shown in Table 1, there are three major types of eating disorders. According to the *Diagnostic and Statistical Manual of Mental Disorders (DSM-5-TR)* published in 2022 [13], anorexia nervosa (AN) can be diagnosed if someone meets the following criteria: restricted energy intake leading to significantly low body weight, fear of gaining weight, and inaccurately perceiving their body weight or shape. The criteria for bulimia nervosa (BN) include repeated binge eating (eating a large amount of food within a short period of time) and repeated inappropriate compensatory behaviors such as vomiting, over-exercising, and use of laxatives. Both behaviors occur at least once a week for 3 months. The criteria for binge eating disorder (BED) include binge eating with marked distress, no inappropriate compensatory behaviors, and occurring at least once a week for 3 months. There are several changes compared to an earlier version of DSM-4: First, the binge eating disorder became an official, “real” diagnosis. In DSM-4, it was under the category of Eating Disorders Not Otherwise Specified. Second, the diagnosis criterion of binge eating for BN and BED is at least once a week for three months. In DSM-4, it was at least twice a week for 6 months. These changes indicate the importance and severity of BED.

Another set of diagnostic criteria used widely is the *WHO International Classification of Diseases and Related Health Problems (ICD-11)* used globally by 194 countries [14]. The newest version was released in Jan 2022. For the most part, the criteria of ICD-11 are consistent with DSM-5-TR. Several differences do exist. For example, the binge eating and inappropriate compensatory behaviors are classified as “at least once a week for one month” in ICD-11 as compared to “at least once a week for three months”. From this aspect, the ICD-11 criteria are less stringent, while DSM-5-TR is relatively stricter for clinical diagnosis.

It is worth pointing out that the term “eating disorders” is different from the term “disordered eating”. “Eating disorders” refers to diagnosed diseases identified by defined signs and symptoms with shown health complications. Disordered eating refers to behaviors such as self-induced vomiting, dieting for weight loss, binge eating, fasting, excessive exercise, and laxative or diuretic use. These behaviors cannot be categorized as complete diseases; however, they must be closely evaluated because they can evolve into true eating disorders.

Eating disorders are very complex illnesses. We still do not fully understand what causes them. Like other complex diseases, many things are involved [15]. Some problems are psychological such as lower self-esteem, depression, anxiety, feeling of loss of control or worthlessness, identity concerns, family communication problems, inability to cope with emotions, or perfectionism. Sociological factors include messages that indicate “to be happy and successful must be thin”, dysfunctional families, sexual or physical abuse, domineering coaches, or controlling relationship. Other biological factors can be genetic factors, altered function of some hormones or neurotransmitters such as serotonin, norepinephrine, cortisol, neuropeptide-Y, peptide-YY, cholecystokinin-CCK, GABA-B, and newly discovered microbiome area [16,17,18].

## 3. Impact of COVID-19 on Eating Disorders

The COVID-19 pandemic has led to the development and exacerbation of eating disorders. In one study [19], the eating disorder inpatient admission cases from 2018 to February 2021 were examined among adolescents and young adults (8–26 years old) in the US. Prior to the pandemic, the admission rates were stable at about 10%. After the pandemic, the admission rate has increased over time to about 20% in January 2021. In addition, during the COVID-19 period, there was more restricting eating, binge eating, and increased over-exercising behaviors reported in eating disorder patients in Australia [20].

During COVID-19, eating disorders increased with the social isolation from the disruption of daily activities, attending less treatment sessions, and increasing anxiety and stress levels [21,22,23]. In addition, there were many unfavorable changes noted in the eating habits and dietary environments. For example, there were increased snacking and emotional eating with boredom being associated with snacking [24,25,26,27]. There was also increased eating in confinement. More than half of respondents in an Italian study reported that they were eating more during confinement; about 20% reported gaining weight, and 42.7% attributed their increase in food consumption to higher anxiety levels [28]. In some cultures, health policy implemented as a counter measure against COVID-19 such as “silent-eating rule” or “mokusyoku rule” may negatively impact the mental health of children [29]. These changes all trigger the development of new eating disorders and make the existing eating disorders worse. Indirectly, COVID-19 itself increased mortality rates and made the overall mental health digress [30].

## 4. Comorbidities of Eating Disorders

### 4.1. Psychiatric Disorders

Patients with eating disorders exhibit high rates of psychiatric comorbidities [31]. The most prevalent psychiatric comorbidities include mood and anxiety disorders, alcohol and substance abuse, and bipolar disorder [32]. Lifetime suicidality rates also increased by 3–5 times in adolescent patients with eating disorders [33]. The morbidities intensify eating disorder symptoms and impact treatment regarding recovery, level of care, and drop-out rates. Therefore, treatment should address co-existing conditions and eating disorders.

### 4.2. Obesity

Besides psychiatric comorbidities, there are numerous medical comorbidities associated with eating disorders including cancers, endocrine, circulatory, pulmonary, gastrointestinal, musculoskeletal, hematologic, and neurologic diseases [34]. Among them, this paper will focus on obesity, diabetes, and metabolic syndrome.

Obesity and eating disorders have a bidirectional impact. On one hand, there are higher eating disorder risks in overweight or obese individuals, especially for BED. Some studies conducted in different populations all show similar results [35,36,37,38]. For example, adolescents who are overweight or obese were at increased risk for developing eating disorders (28.2% higher with overweight and 33% higher with obese) than those who are not overweight or obese [35]. College students with obesity compared to those with a healthy weight had higher rates of clinical and sub-clinical binge eating disorder and lower rates of bulimia nervosa [36]. In a review paper, individuals seeking weight control treatment had a prevalence of 30% for eating disorders [37]. Among bariatric surgery patients, those who meet clinical diagnosis for BED range from 4.2% to 47% depending on the methods of assessment [38].

On the other hand, there is a higher obesity rate observed among BED and BN patients. It was reported that 30% of female patients with eating disorders had lifetime obesity [39]. Those with BED are 3–6 times more likely to be obese than individuals without BED [6]. Consistently, in another study, the lifetime obesity prevalence is close to 90% in BED patients [40].

### 4.3. Diabetes

Eating disorders and diabetes also have a bi-directional impact. Studies reported that eating disorders are more prevalent in people with type 1 diabetes mellitus (T1DM) than in controls [41,42,43]. Teenager girls with T1DM are twice as likely to have eating disorders than those without T1DM [42]. In another study, T1DM patients often experience diabulimia, which is the restriction and removal of insulin, due to the fear of gaining weight with insulin treatment [43]. Moreover, 40–60% of the prevalence of eating disorders is associated with heritability, indicating that it runs in the family and resulting in a genetic link [41].

Additionally, in a population of patients with T1DM and eating disorders, 93.8% reported being diagnosed with T1DM before their eating disorder diagnosis [44]. This suggests that T1DM patients may have experienced more psychopathological factors such as feeling overwhelmed by adhering to healthy eating which leads to behaviors such as bingeing, vomiting, or withholding insulin among eating disorder-prone individuals.

For type 2 diabetes mellitus (T2DM) patients, the eating disorder prevalence rates have ranged widely from 1.4% to 25%, possibly due to differences in criteria of sample characteristics and the setting [45]. Disordered eating behaviors affect up to 40% of patients with T2DM [46]. The comorbid patients have a higher BMI compared to T2DM patients without BED [46]. Individuals with T2DM are more likely to report poorer self-efficacy for following the dietary recommendations set by experts, instead of alternating between BED and night-eating syndrome [47].

In addition, in a community-based sample of adolescents, there were higher rates of disordered eating behaviors in participants with diabetes compared to their peers without diabetes [48]. Specially, those behaviors include increased rates of self-induced vomiting (diabetes 19.2% vs. no diabetes 3.3%; *p* < 0.001) and laxative use (diabetes 15.4% vs. no diabetes 2.1%; *p* < 0.001). Finally, 17% of those with diabetes reported frequent insulin restriction, i.e., diabulimia (≥once per week) [48].

Similar to the report with T1DM patients, T2DM patients indicated that their diabetes diagnosis preceded the eating disorder diagnosis [49]. Patients with comorbidities of diabetes and eating disorders usually require a multidisciplinary treatment approach. In fact, when eating disorder treatment and diabetes management were synergistically integrated, they experienced improvements in both binge eating and glycemic outcomes [49].

### 4.4. Metabolic Syndrome

Metabolic syndrome is a group of risk factors for diabetes and CVD including abdominal fat, high blood pressure, high blood sugar, and hyperlipidemia. Metabolic syndrome is a comorbidity of some eating disorders. For example, adolescent participants with metabolic syndrome were twice as likely to have abnormalities in eating behavior, e.g., restrictive eating or emotional eating than in patients without metabolic syndrome [50].

In obese patients with BED, almost half of the participants met the criteria for metabolic syndrome in two studies [51,52]. Moreover, it is consistently found that men are more likely to meet the metabolic syndrome than women [53]. Therefore, male eating disorder patients may experience more adverse clinical consequences than females.

## 5. Mechanisms Underlying the Comorbidities

Overall, the risks of metabolic diseases vary by eating disorder type according to a recent review paper [54]. For AN, there is no sufficient evidence of the risk of metabolic syndrome. There is a decreased risk for obesity and possibly decreased risk for T2DM. The major explanation is the genetic correlations. The research found significant single-nucleotide-polymorphism (SNP)-based genetic correlations of AN with lower BMI, less insulin resistance, absence of obesity, lower T2DM, and increased high-density lipoprotein (HDL) cholesterol. For BN, the current data is conflicting. BN possibly increases the risk of metabolic syndrome, obesity, and T2DM. The effect is largely accounted for by body weight. For BED, data show the increased risk of metabolic syndrome, obesity, and T2DM. Some effects are largely accounted for by body weight, though there are some other effects independent of body weight including binge eating itself, factors associated with co-occurring psychiatric disorders, and genetic factors.

Understanding the biological mechanisms underlying eating disorders and their comorbidities with metabolic diseases is important for developing novel, highly effective treatments. Some mechanisms have been proposed at either individual or environmental levels [55,56]. At the individual level, possible factors include: FTO (fat mass and obesity-associated) gene; high body dissatisfaction and emotional dysregulation; specific neuro circuit such as hedonic eating, and the dysregulation of the neuroendocrine system, hormones, and neurotransmitters (serotonin, neuropeptide Y, leptin, ghrelin). More recent research also suggested some links between eating disorders and obesity through the gut microbiome [57,58,59]. In many studies, the reduction in microbial alpha diversity and gut dysbiosis have been observed in obese individuals. With limited research, reduced alpha diversity has also been shown in AN patients at both admission and discharge. Gut dysbiosis was reported in AN patients and probiotics helped with the recovery of AN patients [59,60]. At the environmental level, thin body images from social media, overall obesity stigma, and the availability of easy palatable food may all contribute to the comorbidity of eating disorder and obesity [55,56].

## 6. Treatment and Clinical Guidelines of Eating Disorders

Effective treatment is very important for full recovery. Currently, in the US, the treatment for eating disorders typically consists of a combination of the management of medical complications, psychosocial/psychiatric therapy, and nutritional rehabilitation [13,61,62]. Some typical psychotherapies include cognitive behavioral therapy (which focuses on the dysfunctional thoughts and behaviors involved in an eating disorder) and family-based therapy (which is an intensive outpatient program for children and teens involving the whole family) [63]. In family-based therapy, the core is centered around family meals, empowering parents to make decisions, and providing nutrient-dense meals [63]. Medications include antidepressants, antiepileptic medications, anti-obesity medications, and stimulant medications. Currently, lisdexamfetamine is the only approved drug by the US Food and Drug Administration for treating BED and fluoxetine is the only approved drug for treating BN [64,65].

The treatment of eating disorders also requires professional help from a multidisciplinary group of experts: a mental health clinician, a registered dietitian, a medical clinician, and a nurse. The treatment will usually start with a primary doctor who will refer to a mental health professional for psychological counseling; a registered dietitian for nutrition counseling, e.g., restoration of energy and nutrient intake such as zinc, vitamin D, copper, for the AN patient [66]; dental for eating disorder problems with teeth; and involve the family especially if the patient is young [61]. In extreme cases, the patient can be admitted to a hospital for in-patient care.

The treatment goals and approaches are specific for types of eating disorders [61,62]. Briefly, treatment for AN involves weekly family or individual therapy that assists parents and patients with meal plan. A medical provider will supervise the care and make referrals to a psychologist, mental health provider, and dietitian when needed. BN is usually treated with cognitive behavioral therapy to change negative thoughts, family therapy for involving others especially if a child, and interpersonal psychotherapy to address relationship conflicts. Medications such as antidepressants, nutrition education, and hospitalization if needed. BED treatment will involve cognitive behavior therapy for negative feelings that trigger episodes, interpersonal therapy for relationships, and dialectic behavioral therapy to learn behavioral skills to tolerate stress and regulate emotions. BED will also incorporate the medications topomax and antidepressants along a weight-loss program.

There are also evidence-based clinical guidelines for eating disorders in other countries. In a review paper, nine guidelines were compared, and the paper found many consistent recommendations but also many notable differences [67]. For example, all guidelines recommend a multidiscipline approach; the psychological intervention, e.g., cognitive behavioral therapy, is the first line of treatment. However, nutritional counseling was not included in many countries. Selective serotonin reuptake inhibitors (SSRIs) are usually recommended in most guidelines. No other medications are usually recommended since no strong evidence of effective medications for eating disorders has been identified yet. In China, treatment seems to focus more on the physical than the psychological [68]. For example, the gastroenterologist was referred for treatment rather than mental health provider. There seemed to be a stigma attached to having a mental issue instead of a physical problem.

## 7. Conclusions

Eating disorders are not a choice. They are serious mental and physical illnesses that involve complex and damaging relationships with food, eating, exercise, and body image. The comorbidities of eating disorders with metabolic diseases present new clinical and public health challenges which deserve more attention and further research. Early detection and intervention are important for the treatment of eating disorders.

## Figures and Tables

**Table 1:** Definition of major eating disorders [13,14] *.

	DSM-5-TR	ICD-11
Anorexia nervosa	Persistent energy intake restriction leading to significantly low body weight Intense fear of gaining weight or of becoming fat A disturbance in self-perceived weight or shape	Significantly low body weight or rapid weight loss A persistent pattern of restrictive eating The person’s body weight/shape is inaccurately perceived
Bulimia nervosa	Recurrent episode of binge eating Recurrent use of inappropriate compensatory behaviors to prevent weight gain Occurred at least once a week for three months	Frequent, recurrent episode of binge eating Repeated inappropriate compensatory behaviors to prevent weight gain Excessive preoccupation with body weight and shape Marked distress about binge eating Occurred at least once a week for one month
Binge eating disorder	Recurrent episode of binge eating Marked distress regarding binge eating Occurred at least once a week for three months	Frequent, recurrent episode of binge eating Excessive preoccupation with body weight and shape Marked distress about binge eating Occurred at least once a week for one month

* Adapted from *DSM-5-TR: Diagnostic and Statistical Manual of Mental Disorders—5th edition-text revision; ICD-11: International Classification of Diseases and Related Health Problems—11th edition.*

## Data Availability

Not applicable.
